# COVID-19 information exposure in digital media and implications for employees in the health care sector: findings from an online survey

**DOI:** 10.31744/einstein_journal/2020AO6127

**Published:** 2020-11-25

**Authors:** Paulo Rodrigo Bazán, Raymundo Machado de Azevedo, Julia Abou Dias, Vanessa Gil Salvatierra, Liana Guerra Sanches, Shirley Silva Lacerda, Edson Amaro, Elisa Harumi Kozasa, Joana Bisol Balardin

**Affiliations:** 1 Hospital Israelita Albert Einstein São PauloSP Brazil Hospital Israelita Albert Einstein, São Paulo, SP, Brazil.

**Keywords:** COVID-19, Coronavirus infections, Social media, Information seeking behavior, Psychological distress, Cross-sectional studies, Survey and questionnaires

## Abstract

**Objective:**

To estimate coronavirus disease 2019-related information consumption and related implications for health care professionals (medical and nonmedical personnel) during the pandemic.

**Methods:**

A cross-sectional on-line survey was distributed to employees of a major health care institution located in São Paulo, Brazil between April 3 and April 10, 2020. Data were analyzed using descriptive statistics.

**Results:**

The sample comprised 2,646 respondents. Most participants (44.4%) reported excessive or almost excessive access to information about the novel coronavirus and 67.6% reported having increased their average time spent on social media. When asked how frequently they consider it was easy to determine the reliability of information, “sometimes” corresponded to 43.2% of the answers in contrast to 14.6% responding “always”. Answers related to potential signs of information overload associated with the pandemic indicated that 31% of respondents felt stressed by the amount of information they had to keep up with almost every day or always. Overall, 80.0% of respondents reported having experienced at least one of the following symptoms: headache, eye twitching, restlessness or sleeping difficulty. The frequency of symptoms was higher among participants with a more negative information processing style regarding when dealing with large volumes of information relative to those with a positive information processing style. Likewise, symptoms were more frequently reported by participants who had increased their social media access relative to those reporting reduced access during the pandemic.

**Conclusion:**

Our survey provides a description of how health professionals consume COVID-19 related information during the pandemic, and suggests that excessive information exposure and high processing demands may impose psychological distress and affect mental health.

## INTRODUCTION

The coronavirus disease 2019 (COVID-19) pandemic has been extensively covered by traditional and social media. The 24/7 TV and radio broadcasting services and the wide access to mobile technologies have allowed an unprecedented number of people to be quickly and regularly updated with the crisis. This massive delivery of fast changing information about scientific aspects of the disease and about its implications on everyday life places high demands on people's abilities to handle this information.^(^^1^^)^

Particularly for those individuals working at the healthcare sector, the information era imposes great challenges for keeping up-to-date with the relevant data,^(^^2^^)^ and these challenges have gained unprecedented proportions during the current moment. Recent meta-analyses suggest health professionals are prone to mental health issues during the COVID-19 pandemic,^(^^3^^–^^5^^)^ although the role of information consumption still needs further evaluation. Therefore, the COVID-19 pandemic is an opportunity to further understand health workers' media consumption preferences, information seeking behaviors and its consequences for mental health.

Repeated exposure to news related to the current coronavirus outbreak may lead to psychological distress.^(^^6^^)^ The idea that media exposure is associated with psychological well-being is not new and has been suggested during previous health crises of similar proportions. For example, during the H1N1 outbreak, increased information-related uncertainty and feelings of uncontrollability were associated with higher anxiety levels.^(^^7^^)^ Likewise, a survey conducted with a nationally representative sample of United States residents during the 2014 Ebola outbreak revealed associations between greater exposure to Ebola-related news and increased levels of distress, worry and functional impairment.^(^^8^^)^ Understanding the emotional impact of media coverage on health professionals during disease outbreaks can help design better communication policies, and interventions aimed to promote psychological well-being in the work environment.

## OBJECTIVE

This survey-based study was designed to quickly estimate coronavirus disease 2019-related information consumption, and its implications among health care professionals working at a major healthcare institution located in São Paulo, Brazil during the COVID-19 pandemic. Sources, types and volume of information consumed by health care professionals, their feelings regarding information processing demands imposed by the pandemic and self-reported symptoms associated with psychological distress were specifically addressed.

## METHODS

### Participants

An online cross-sectional survey was distributed to all employees (approximately 14,000 individuals) of a major health care institution located in São Paulo, Brazil, between April 3 and April 10, 2020. All employees were invited to participate, including frontline workers ( *e.g.,* those delivering direct care to patients, such as physicians, nurses and allied health professionals) and nonmedical personnel ( *e.g.,* researchers, technicians, managers, administrative staff and maintenance personnel). Participants were informed about survey purposes (including potential risks and benefits) and granted their consent by electronic means. The study was approved by the Brazilian National Research Ethics Commission (CONEP; number 3.944.446, CAAE: 30179320.0.0000.0071).

### Survey development and distribution

The survey was provided via an institutional e-mail sent to all employees on 2020, April 3 (Friday). A Workplace post and an e-banner published on April 9, 2020 on the intranet were used as remainders. The survey expiration date (April 10, 2020) was informed in all communication material. Aside from demographic data, the survey comprised 15 items divided into eight questions ( Supplementary materials S1 and S2 for the original and translated versions of the questionnaire). Participants were asked about the type ( *i.e.,* epidemiology, symptoms, preventive measures and treatment), source ( *i.e.,* social media channels, television, radio and e-mail) and volume ( *i.e.,* hours of media consumption per day) of COVID-19-related information they had accessed in the previous week.

Feelings related to information processing demands during the pandemic were investigated using questions adapted from a previous United States national survey about information overload.^(^^9^^)^ A 1-to-5 Likert-type scale (1 corresponds to never and 5 to always) was used to rate, with regard to COVID-19-related information, how often in the previous week participants: felt information reliability was is easy to determine; felt stressed by the load of information they had to keep up with; felt the institution and/or professional role demanded excessive gathering of information in order to deal with the current situation; felt having much information contributed to decision making; felt confident in their ability to use the Internet and other means of communication to handle information-related demands in their life; felt the information they need was easy to find and kept consuming information in spite of feeling they had reached their limits. Other questions addressed participants' physical complaints and behavioral adjustments. All questions were mandatory. The REDCap platform was used to administer the survey.

Survey development was informed by findings of a small pilot study with 7 participants (six females) conducted to test question understandability and acceptability. Individual qualitative responses extracted from the pilot study were encoded to help refine questionnaire wording and layout. A consensus meeting with authors of the study was held to prepare the final version of the questionnaire.

### Data analysis

Data analysis was based on descriptive statistics and carried out using R software ( https://www.r-project.org/ ) and the following packages: tidyverse, stringr, ggthemes, ggalt, ggstance, here, lemon, table 1 , gridExtra, ggpubr, factoextra, tm, SnowballC, wordcloud and RColorBrewer.

**Table 1:** Demographic characteristics of survey respondents

Demographic variable		Participants n (%)
Sex		
	Female	2,066 (78.1)
	Male	580 (21.9)
Age groups, years		
	<25	125 (4.7)
	25-34	890 (33.6)
	35-44	1132 (42.8)
	45-54	399 (15.1)
	55-64	94 (3.6)
	≥65	6 (0.2)
Level of education		
	Primary education, incomplete	1 (0.0)
	Primary education, complete/secondary education, incomplete	18 (0.7)
	Secondary education, complete/higher education, incomplete	46 (1.7)
	Higher education, complete/undergraduate studies, incomplete	936 (35.4)
	Undergraduate degree	656 (24.8)
	MBA	824 (31.1)
	MSc	114 (4.3)
	PhD	51 (1.9)
Monthly income *		
	0-1	33 (1.2)
	1-3	728 (27.5)
	3-6	805 (30.4)
	6-9	437 (16.5)
	9-2	198 (7.5)
	12-15	135 (5.1)
	15 or more	310 (11.7)
Contact with COVID-19 at work		
	No	568 (21.5)
	Yes	2,078 (78.5)

* Brazilian minimum wage at the moment the study was carried out: R$1,045.00.

A total of 3,217 responses were received within one week of questionnaire distribution. Participants who had not answered all questions or duplicated answers were excluded. Participants providing inconsistent answers (selection of “No information”/“No symptom”/“I don't know/I would rather not respond” or any other alternative allowing for multiple answers) were also excluded. The final sample comprised completed 2,646 surveys.

The percentages of respondents by gender, age group, educational level, monthly income and history of contact with COVID-19 at work (yes or no) were calculated in order to characterize the sample. The percentage of respondents who selected values greater than 75 in an online version of a zero (none) to one hundred (excessive) analogue scale was used to determine the amount accessed COVID-19-related information. Participants' access to social media was determined by the difference between time spent on social media per day during the previous week and before the pandemic, as measured using online versions of zero to 24 hours analogue scales. One question designed to inquire about reduction of access to COVID-19-related information in the previous week and reasons for doing so (in positive cases) was also used.

Participants' feelings about the volume of information obtained in the previous week were described according to the percentage of selection of alternatives for each of the seven items rated using 1-to-5 Likert-type scales. The percentage of participants reporting symptoms of psychological distress was also calculated. Assessment of such symptoms was thought to be a less subjective way of examining information exposure effects for deeper analysis of results. To further investigate relations between individual information processing style differences when dealing with information and psychological distress symptoms, respondents were split into two subgroups according to answers given to question 3 ( Supplementary materials S1 and S2 ). One group comprised participants with a more positive style (those who selected “Having a lot of information makes my life easier” and/or “I like to have access to as much information as possible”) whereas the other comprised those with a more negative style (those who selected “I feel overloaded when I have access to too much information” and/or “Having a lot of information makes my life seem more complex”). Participants who answered “I don't know/I would rather not answer” or selected both answers were not included in any of the subgroups.

Relations between frequency of distress symptoms and changes in social media access behavior were also examined. Participants who reported having increased or reduced their access to social media in the previous week were also split into two groups. The frequency of each distress symptom was compared between the different social media access subgroups (increased *versus* reduced) and also between different information processing style subgroups (positive *versus* negative). The 95% confidence intervals were provided for differences in proportions.

A word cloud using terms extracted from answers given to the open-ended question about “other symptoms” was created for further analysis of distress symptoms. A custom script was used to eliminate punctuation marks and accents and to convert plural words into their singular form according to the most common Portuguese plural rules (there are exceptions). Common stop words, conjunctions, adverbs and numerals were then excluded. Remaining words were listed according to frequency of use and examined for typos. Verbs, adjectives and nouns with the same meaning ( *e.g* ., “stress” and “stressed”, “anxiety” and “anxious”) were clustered. Following these adjustments, word frequency was recalculated and those used two or more times selected to create the word cloud. Words were translated into English for publication purposes. However, the original version of the word cloud in the Portuguese language has been provided in the form of supplementary material S3 . Complete data and codes may be accessed upon request sent to the corresponding author and with due approval by the ethics committee.

## RESULTS

### Demographic data

From April 3 to 10, 2020, a total of 2,646 surveys were completed (approximately 19% response rate). Demographic characteristics of the sample are shown in table 1 .

The median age of participants was 37 years (range: 19-74 years). Among the total respondents, 2,066 (78.1%) were women, 989 (37.4%) had a graduate degree, and 1,304 (49.3%) were frontline staff. Most respondents (2,078, 78.5%) reported having contact with confirmed or suspected COVID-19 cases at work, and 756 (28.6%) reported changing their work routine in response to COVID-19. The most common change was to partly work remotely from home (414 people, 15.6%).

### Sources, types and volume of COVID-19-related information

Most respondents (90.1%) accessed COVID-19 information through traditional media channels (TV/radio), followed by WhatsApp (73.0%), word of mouth (57.0%), e-mail (54.4%), Workplace (47.7%), Facebook (47.2%), Instagram (40.4%), YouTube (22.1%), others (10.8%), and Twitter (7.0%). When asked about specific types of information they had received, the most common information was the number of confirmed cases (received by 96.3%), followed closely by prevention measures (94.9%), number of deaths due to COVID-19 (94.4%), and symptoms of COVID-19 (91.0%). Information on possible treatment was reported by 72.9%, while 8.8% reported other pieces of information, and 0.2% reported not having received any information about COVID-19.

Next, efforts were made to characterize the volume of COVID-19-related information respondents had accessed and whether they have changed their social media access behavior during the pandemic. Approximately 44.4% of respondents reported excessive access (scores higher than 75 in a zero to one hundred scale, supplementary materials S1 and S2 ) of COVID-19-related information. Most participants (67.6%) had also increased their daily media consumption (mean increase of 2.46 hours, standard deviation of 4.35 hours) relative to their regular access prior to the pandemic. In spite of the average increase in social media consumption, 45.5% participants reported having reduced their access to COVID-19-related information in the previous week. This was mostly due to repetitive information (77.7%). However, tiredness (59.54%) and fear regarding information content (19.8%) were also reported, among other reasons (14.0%).

### Feelings related to information processing demands during the pandemic

**Figure 1:**
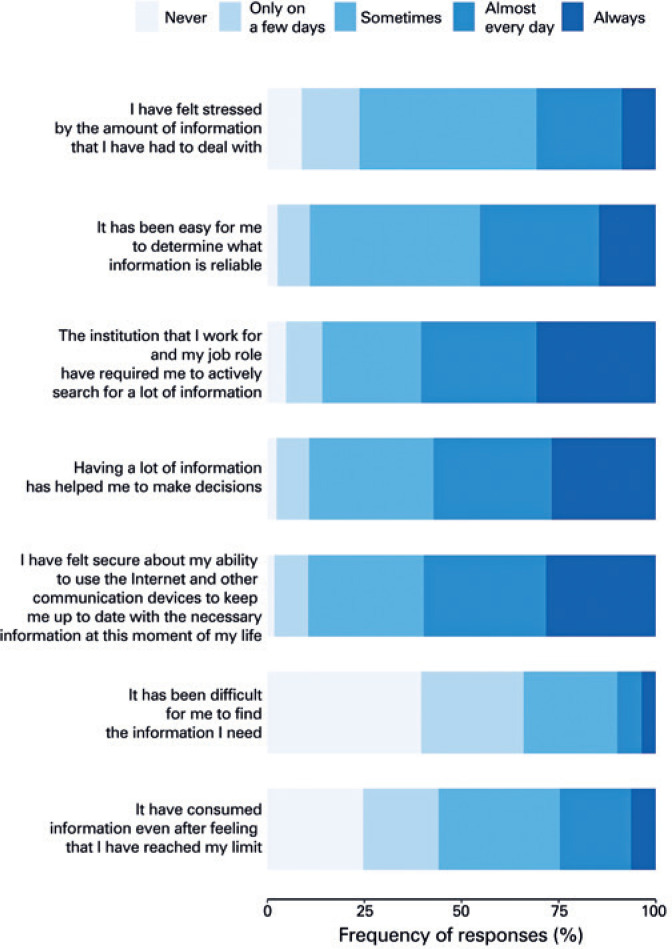
Perception and feelings associated with COVID-19-related information. Frequency of responses to each question on a Likert scale, presented as horizontal cumulative bar plots

With regard to perceptions and feelings associated with COVID-19-related information ( Figure 1 ), many participants reported feeling stressed due to the amount of information they had to keep up with (always or almost every day: 30.7%). Nevertheless, 24.7% of respondents kept consuming information even after having reached their limit (always or almost always). When asked about how easy it was to determine the reliability of COVID-19-related information, most participants answered it was sometimes easy (43.8%). Most participants reported having no difficulties to find the information they needed (never: 39.6%; at least sometimes: 34.0%) and feeling confident about their own ability to find the information they needed most of the time (always or almost every day: 59.7%). Having obtained the information required, most respondents felt it was helpful to make decisions (always or almost every day: 57.1%). Also, most of them reported being required by their job or institution to look for information about COVID-19 (always and almost every day: 60.4%).

### Complaints related to psychological distress

**Figure 2:**
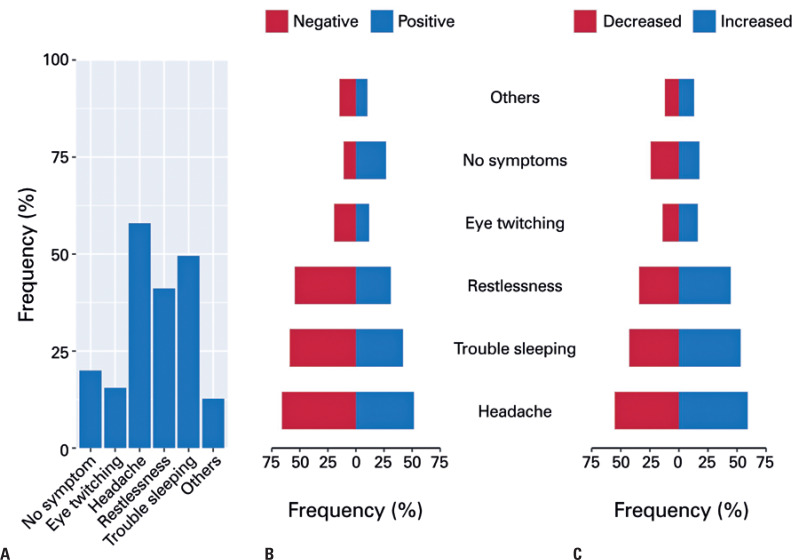
Prevalence of psychological distress symptoms. (A) Bar plots displaying the frequency of symptoms among all participants; (B) frequency of symptoms according to positive or negative information processing style; (C) frequency of symptoms according to increase or reduction of the time spent on social media ( *i.e.* , hours of media consumption per day) in the week prior to the survey relative to prior to the pandemic

Participants were asked whether they had experienced symptoms associated with psychological distress in the previous week. More than half of participants reported having experienced headache (57.9%) and almost half had had trouble sleeping (49.5%), among other symptoms ( Figure 2 A). Analysis of subgroups of processing styles when dealing with information overload revealed higher frequency of psychological distress symptoms among participants with a negative relative to those with a positive style ( Figure 2 B; confidence intervals for differences in proportions: headache = 10.38–18.71; trouble sleeping = 12.58%–21.05%; restlessness = 19.22%–27.54%; eye twitching = 4.64%–10.99%; no symptoms = −12.68%– −19.12%; others = 1.44%–7.21%). The frequency of psychological distress symptoms was also higher among participants reporting increased social media access during the pandemic relative to those reporting reduction, except for “eye-twitching” and “other symptoms” ( Figure 2 C; confidence intervals for differences in proportions: headache = 0.01%–8.25%; trouble sleeping = 6.14%–14.39%; restlessness = 6.29%–14.31%; eye-twitching = −0.79%–5.15%; no symptoms = −2.96%– −9.89%; others = −1.96%–3.58%).

**Figure 3:**
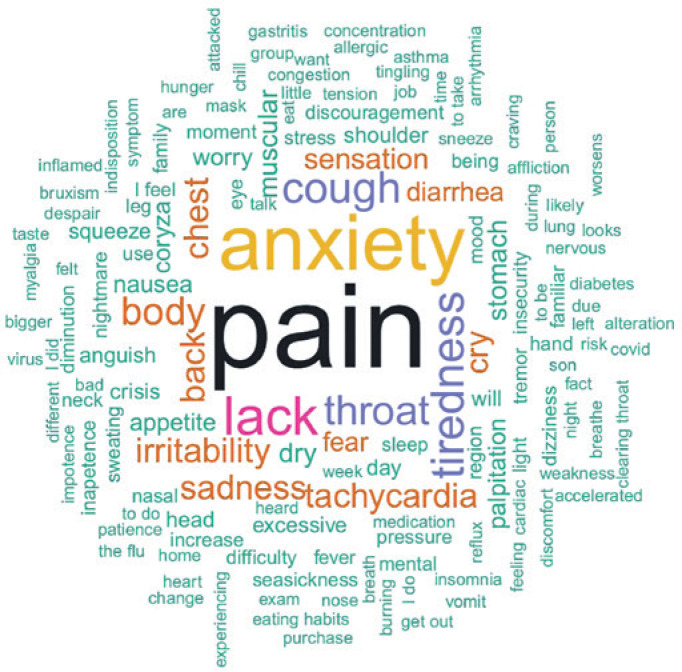
Word cloud used to describe psychological distress symptoms. Word cloud created with words extracted from texts provided by respondents in the field “others”, when inquired about symptoms experienced. Words used only one time were omitted

As regards other symptoms reported in answers to the open-ended question, the most commonly used words were pain (111 times), anxiety (70 times), lack (43 times), tiredness (34 times) and throat (33 times). The word cloud containing words used two or more times is shown in figure 3 . Importantly, the word pain also expresses the idea of “ *ache* ”, given the original Portuguese word “ *do* r” can be translated either way, depending on the context. For example, “headache” means “ *dor de cabeça* ” in Portuguese, whereas “back pain” means “ *dor nas costas* ”. The same applies to for the Portuguese word “ *falta* ”, which can be translated as “lack”, as in “lack of appetite”, or “shortness”, as in “shortness of breath”. The word cloud containing the original words in Portuguese is shown in supplementary material S3 .

## DISCUSSION

This cross-sectional online survey conducted with employees of a major health care institution located in São Paulo, Brazil, included 2,646 respondents and revealed an overall increase in average daily exposure to COVID-19-related information distributed on traditional and social media channels and a corresponding increase in the frequency of psychological distress symptoms. Most participants were female with a median age of 37 (19 to 74) years. Most respondents had a high educational level (undergraduate degree or higher). In summary, this study provided insights into COVID-19-related information consumption behavior among health workers during the pandemic. The findings also suggest excessive information exposure and high information processing demands may impose psychological distress.

Increased levels of daily media consumption reported by most participants in this sample is in keeping with mass communication theories describing increased reliance on media in times of uncertainty and crisis.^(^^10^^)^ Most respondents also reported the use of traditional media channels, such as television, to obtain COVID-19-related information. Interestingly, the second most used channel was WhatsApp, a type of social media widely employed in Brazil. Preferential exposure to channels exhibiting graphic content may also be important for the understanding of health implications of media consumption during disease outbreaks. In the case of the 2013 Boston Marathon bombings, for example, associations between increased exposure to images containing blood and higher levels of posttraumatic stress and fear of the future, 6 months after the incident have been reported.^(^^11^^)^ However, findings of this study revealed that a significant proportion (45.5%) of participants deliberately reduced their access to COVID-19-related information due to factors such as repetitive information and tiredness, suggesting self-control strategies play an important role in the regulation of information consumption behavior.^(^^12^^)^

Answers related to potential signs of information overload associated with the pandemic indicated that approximately one-third of respondents felt stressed by the amount of information they had to keep up with almost every day or always. Overall, more than half of respondents experienced at least one psychological distress symptom (headache, eye twitching, restlessness or trouble sleeping) and the frequency of symptoms was higher among participants with a negative bias towards excessive information processing demands. Ache/pain and anxiety were the most common words in the answers provided to the open-ended question related to symptoms of psychological distress. Also, psychological distress symptoms were more common among participants reporting increased use of social media. Wide media coverage and factors such as the long-lasting rise in the number of COVD-19 infection cases, the overwhelming workload and insufficient personal protective equipment were thought to contribute to mental health outcomes among Chinese health care workers during the COVID-19 crisis. Higher rates of insomnia were documented among medical staff who dedicated more than 5 hours to reading COVID-19-related news.^(^^13^^)^ Positive associations between high prevalence of mental health problems and social media exposure have also been reported in the Chinese population,^(^^14^^)^ whereas longer time spent reading COVID-19-related information was associated with higher levels of anxiety in the Russian population.^(^^15^^)^ These findings support to the hypothesis that repeated media exposure during the current coronavirus outbreak may be associated with psychological distress.^(^^3^^)^ However findings of this survey indicate that individual information processing styles differences when dealing with information overload may modulate psychological distress associated with information exposure, given psychological distress symptoms were more common among participants who perceived information overload as stressful and/or complex.

Although spending more time consuming COVID-19 information has been related to psychological distress, having accurate and up-date health information seems to be a protective factor.^(^^16^^)^ In this study, most participants reported having no difficulty finding the information they needed (39.6%), although difficulties were reported by a significant percentage (34.0%) at least sometimes. Also, the fact that most participants felt information reliability was sometimes easy to determine indicates it was sometimes not easy, even for employees of a major health care institution. Therefore, the potential protective effect may have been mitigated in this sample.

Limitations of our study include the use of a convenience sample comprising primarily women (most employees of the health care institution selected are female), low response rate (19%) and unknown psychometric validity of the survey (the questionnaire administered has not been validated yet). Another potential limitation is the change in media access behavior among participants over the course of the pandemic, as shown by the percentage of respondents who reported having reduced their access in the week of the survey. It should be noted that this survey was launched 23 days after declaration of pandemic by the World Health Organization (March 11, 2020), and 10 days after the official implementation of quarantine measures in São Paulo (March 24, 2020). Therefore, further studies are warranted to investigate changes in media access behaviors during the pandemic. Nevertheless, limitations aside, findings of this survey may contribute to the design of communication policies and interventions aimed at promoting psychological well-being in the work environment.

## CONCLUSION

This study provided a description of COVI-19-related information consumption behavior among health care professionals during the pandemic. Findings suggest excessive information exposure and high information processing demands may impose psychological distress symptoms.
