# Supplementary material for: COVID-19 information exposure in digital media and implications for employees in the health care sector: findings from an online survey
Source: Einstein (Sao Paulo). 2020 Nov 25;18:eAO6127. doi: 10.31744/einstein_journal/2020AO6127 (PMC7690931; doi:10.31744/einstein_journal/2020AO6127)
Supplement: Translation

# Translation: Exposição às informações sobre COVID-19 em mídias digitais e suas implicações para funcionários do setor de saúde: resultados de uma pesquisa *on-line*

**Authors:** Paulo Rodrigo Bazán, Raymundo Machado de Azevedo, Julia Abou Dias, Vanessa Gil Salvatierra, Liana Guerra Sanches, Shirley Silva Lacerda, Edson Amaro, Elisa Harumi Kozasa, Joana Bisol Balardin

**Affiliations:** 1 São PauloSP Brasil Hospital Israelita Albert Einstein, São Paulo, SP, Brasil.

**Keywords:** COVID-19, Infecções por coronavírus, Mídias sociais, Comportamento de busca de informações, Sofrimento psicológico, Estudos transversais, Inquéritos e questionários

## Abstract

**Objetivo:**

Estimar o consumo de informações relacionadas com doença do novo coronavírus e seus efeitos em profissionais do setor da saúde durante a pandemia.

**Métodos:**

Um questionário *on-line* foi distribuído para funcionários de uma instituição de saúde em São Paulo, Brasil, entre 3 e 10 de abril de 2020. Os dados foram analisados com estatísticas descritivas.

**Resultados:**

Foram incluídos nas análises 2.646 participantes. A maioria (44,4%) reportou ter acessado uma quantidade excessiva ou próxima de excessiva sobre o novo coronavírus, e 67,6% reportaram ter aumentado seu tempo médio em mídias sociais. Quando perguntados se era fácil determinar o que era informação confiável, 43,2% responderam “às vezes”, comparados com 14,6% que responderam “sempre”. Sobre os possíveis sinais de sobrecarga de informação associada com a pandemia, 31% sempre ou quase todos os dias se sentiram estressados com a quantidade de informações que tinham que acompanhar. Entre os respondentes, 80,0% reportaram sentir pelo menos um sintoma como dor de cabeça, espasmos oculares, inquietação ou dificuldade para dormir. Participantes com um estilo mais negativo de lidar com muitas informações também reportaram maior proporção de sintomas que os participantes com estilo positivo. De forma semelhante, participantes que aumentaram seu acesso a mídias sociais reportaram maior proporção de sintomas do que os que diminuíram seu acesso durante a pandemia.

**Conclusão:**

Nossa pesquisa fornece uma descrição de como os indivíduos consomem informações relacionadas com a doença do novo coronavirus durante a pandemia e sugere que a exposição a uma quantidade excessiva de informações e as elevadas demandas podem impor sofrimento psicológico e afetar a saúde mental.

## INTRODUÇÃO

A pandemia causada pelo novo coronavírus (COVID-19) foi amplamente coberta pelas mídias tradicional e social. A transmissão por rádio e televisão durante 24 horas, todos os dias da semana, e o amplo acesso a tecnologias móveis permitiram que um número sem precedentes de pessoas recebesse atualizações a respeito da crise, de forma rápida e regular. A enorme divulgação e a rápida mudança das informações referentes aos aspectos científicos da doença e suas implicações na vida diária impuseram alta demanda à habilidade das pessoas de lidar com as informações.^(^^1^^)^

A era da informação impõe grandes desafios para se estar atualizado em relação aos dados relevantes, principalmente entre os profissionais de saúde,^(^^2^^)^e tais desafios adquiriram proporções sem precedentes no momento atual. Metanálises recentes sugerem que os profissionais de saúde têm maior tendência ao desenvolvimento de problemas mentais durante a pandemia de COVID-19,^(^^3^^–^^5^^)^embora o papel do consumo de informações ainda requeira investigação mais aprofundada. Dessa forma, a pandemia de COVID-19 se apresenta como uma oportunidade para a melhor compreensão das preferências de consumo de mídia entre os profissionais da saúde, assim como de seus comportamentos de busca de informações e suas respectivas consequências para a saúde mental.

A exposição repetida à mídia relacionada ao surto atual de coronavírus pode ter relação com o sofrimento psicológico.^(^^6^^)^A ideia de que a exposição à mídia tem relação com o bem-estar psicológico não é nova e foi sugerida em crises passadas de saúde de proporções semelhantes. Durante o surto de H1N1, por exemplo, o aumento da incerteza sobre as informações e os sentimentos de descontrole foram associados a níveis mais elevados de ansiedade.^(^^7^^)^De modo semelhante, uma pesquisa realizada com amostra nacionalmente representativa de residentes dos Estados Unidos durante o surto de ebola de 2014 revelou associações entre maior exposição a notícias relacionadas ao ebola e maiores níveis de estresse, preocupação e comprometimento funcional.^(^^8^^)^A compreensão do impacto emocional da cobertura midiática nos profissionais da saúde durantes surtos de doenças pode contribuir para a elaboração de políticas mais adequadas de comunicação e intervenções destinadas a promover o bem-estar psicológico no ambiente de trabalho.

## OBJETIVO

Esta pesquisa teve por objetivo estimar rapidamente o consumo de informações relacionadas à COVID-2019 e suas implicações para os funcionários de uma instituição de saúde localizada em São Paulo, Brasil durante a pandemia. Foram avaliados especificamente as fontes, os tipos e o volume de informações consumidas por esses profissionais, seus sentimentos com relação às demandas de processamento de informações impostas pela pandemia e os sintomas autorrelatados relacionados ao sofrimento psicológico.

## MÉTODOS

### Participantes

Uma pesquisa transversal *on-line* foi distribuída a todos os funcionários (aproximadamente 14 mil pessoas) de uma importante instituição de saúde localizada em São Paulo, entre 3 e 10 de abril de 2020. Todos os funcionários foram convidados a participar, incluindo profissionais da linha de frente (envolvidos no cuidado direto dos pacientes, como médicos, enfermeiros e outros profissionais de saúde) e o pessoal de fora da área clínica (pesquisadores, técnicos, administradores, funcionários administrativos e profissionais da manutenção). Os participantes foram informados quanto aos objetivos da pesquisa (incluindo possíveis riscos e benefícios) e deram seu consentimento por meio eletrônico. Este estudo foi aprovado pela Comissão Nacional de Ética em Pesquisa (CONEP, número 3.944.446, CAAE: 30179320.0.0000.0071).

### Desenvolvimento e distribuição da pesquisa

A pesquisa foi disponibilizada por meio de um *e-mail* institucional enviado a todos os funcionários no dia 3 de abril (sexta-feira) de 2020. Além disso, uma postagem publicada pelo Workplace, no dia 9 de abril de 2020, foi utilizada como lembrete, juntamente de um *e-* *banner* disponibilizado na intranet. A data limite para resposta (10 de abril de 2020) foi especificada em todos os comunicados. Além de dados demográficos, a pesquisa incluiu 15 itens divididos em oito perguntas ( Materiais suplementares S1 e S2 para as versões original e traduzida das perguntas). Foram interrogados o tipo (epidemiologia, sintomas, medidas preventivas e tratamento), as fontes (canais de mídia social, televisão, rádio e *e-mail* ) e o volume (horas de consumo diário de mídia) de informações relacionadas à COVID-19 acessadas pelos participantes na semana anterior.

Os sentimentos relacionados à demanda de processamento de informação durante a pandemia foram avaliados com base em perguntas adaptadas de uma pesquisa anterior sobre sobrecarga de informações aplicada em nível nacional nos Estados Unidos.^(^^9^^)^Para essas perguntas, uma escala tipo Likert de 1 a 5, sendo 1 correspondente a nunca e 5 a sempre, foi utilizada para determinar, no que se refere a informações relacionadas à COVID-19, o quão frequente ao longo da semana anterior os participantes acharam fácil determinar a confiabilidade das informações; sentiram-se estressados com a quantidade de informações para acompanhar; acharam que a instituição e/ou o cargo exigiram coleta excessiva de informações para lidar com a situação presente; acharam que ter muitas informações foi útil na tomada de decisão; sentiram-se confiantes quanto à própria capacidade de usar a Internet ou outro meio de comunicação para lidar com as demandas de informações em suas vidas; acharam fácil encontrar as informações necessárias e continuaram consumindo informações apesar de sentirem ter atingido o limite. Outras perguntas se voltaram para as queixas físicas dos participantes e os ajustes comportamentais. Todas as perguntas eram de resposta obrigatória. A pesquisa foi disponibilizada por meio da plataforma REDCap.

Um pequeno estudo piloto foi realizado para desenvolvimento do questionário. Esse estudo envolveu sete participantes, sendo seis mulheres, que testaram a facilidade de compreensão e a aceitação das perguntas. As respostas individuais qualitativas obtidas no estudo piloto foram codificadas a fim de ajudar a aprimorar a redação e a apresentação do questionário, e houve uma reunião de consenso com os autores do estudo para preparar a versão final.

### Análise dos dados

A análise dos dados foi baseada na estatística descritiva, realizada empregando-se o software R ( https://www.r-project.org/ ) e os seguintes pacotes: tidyverse, stringr, ggthemes, ggalt, ggstance, here, lemon, table 1 , gridExtra, ggpubr, factoextra, tm, SnowballC, wordcloud e RColorBrewer.

**Tabela 1:** Características demográficas dos respondentes

Variável demográfica		Participantes n (%)
Sexo		
	Feminino	2.066 (78,1)
	Masculino	580 (21,9)
Faixas etárias, anos		
	<25	125 (4,7)
	25-34	890 (33,6)
	35-44	1132 (42,8)
	45-54	399 (15,1)
	55-64	94 (3,6)
	≥65	6 (0,2)
Escolaridade		
	Fundamental incompleto	1 (0,0)
	Fundamental completo/ensino médio incompleto	18 (0,7)
	Ensino médio completo/ensino superior incompleto	46 (1,7)
	Ensino superior completo/graduação incompleta	936 (35,4)
	Graduado	656 (24,8)
	MBA	824 (31,1)
	Mestrado	114 (4,3)
	Doutorado	51 (1,9)
Renda mensal *		
	0-1	33 (1,2)
	1-3	728 (27,5)
	3-6	805 (30,4)
	6-9	437 (16,5)
	9-2	198 (7,5)
	12-15	135 (5,1)
	15 ou mais	310 (11,7)
Contato com COVID-19 no ambiente de trabalho		
	Não	568 (21,5)
	Sim	2.078 (78,5)

* Salário mínimo no Brasil o momento da pesquisa: R$1.045,00.

Uma semana após o envio do questionário, 3.217 respostas tinham sido recebidas. Participantes que não responderam a todas as perguntas ou forneceram respostas duplicadas foram excluídos, assim como participantes que forneceram respostas inconsistentes (seleção de “Nenhuma informação”/“Nenhum sintoma”/“Não sei/Prefiro não responder”, ou qualquer outra alternativa que permitisse múltiplas respostas). A amostra final incluiu 2.646 pesquisas completas.

As percentagens de respondentes por gênero, faixa etária, escolaridade, renda mensal e de acordo com o histórico de contato com a COVID-19 no ambiente de trabalho (sim ou não) foram calculadas para caracterização da amostra. A percentagem de respondentes que selecionaram valores acima de 75 na versão eletrônica de uma escala analógica de zero (nenhum) a cem (excessivo) foi utilizada para caracterizar a quantidade de informações acessadas relacionadas à COVID-19. O acesso de mídia social pelos participantes foi determinado de acordo com a diferença entre o tempo diário dispendido com mídia social na semana anterior à pesquisa e antes da pandemia, sendo mensurado por meio de versões *on-line* de escalas analógicas de zero a 24 horas. Foi utilizada também uma pergunta que avaliou a diminuição do acesso a informações relacionadas à COVID-19 na semana anterior e os motivos dessa diminuição (nos casos pertinentes).

Os sentimentos dos participantes a respeito do volume de informações obtido na semana anterior à pesquisa foram descritos com base na percentagem de seleção das alternativas disponíveis para cada um dos sete itens graduados nas escalas do tipo Likert de 1 a 5. A percentagem de participantes que relataram sintomas de sofrimento psicológico também foi calculada. A avaliação desses sintomas foi considerada uma forma menos subjetiva de investigar os efeitos da exposição às informações, permitindo uma exploração mais profunda dos resultados. A fim de investigar mais a fundo a relação entre diferenças individuais de estilo de processamento de informações diante da sobrecarga de informações e os sintomas de sofrimento psicológico, os respondentes foram divididos em dois subgrupos, de acordo com as respostas fornecidas à pergunta 3 ( Materiais suplementares S1 e S2 ). Um grupo foi formado pelos participantes com um estilo mais positivo (os que selecionaram as respostas “Ter muitas informações à disposição facilita a minha vida” e/ou “Gosto de ter o máximo de informação possível”), enquanto o outro foi formado pelos que demonstraram um estilo mais negativo (os que selecionaram as respostas “Sinto-me sobrecarregado quando tenho muitas informações à disposição” e/ou “Ter muitas informações à disposição faz minha vida parecer mais complexa”). Os participantes que responderam “Não sei/Prefiro não responder” ou selecionaram ambas as respostas não foram incluídos em nenhum dos subgrupos.

A relação entre a frequência de sintomas de sofrimento psicológico e as alterações de comportamento de acesso a mídias sociais também foi examinada. Os participantes que relataram aumento ou diminuição do acesso a mídias sociais na semana anterior foram divididos em dois grupos. A frequência de cada sintoma de sofrimento psicológico foi comparada entre os subgrupos de acesso às mídias sociais (aumento ou diminuição) e também entre os subgrupos com diferentes estilos de processamento de informação (positivo ou negativo). Os intervalos de confiança de 95% para as diferenças de proporções foram fornecidos.

Para analisar mais a fundo os sintomas de sofrimento psicológico, foi criada uma nuvem de palavras a partir das respostas fornecidas para a questão aberta sobre “outros sintomas”. Para isso, foi empregado um *script* personalizado. Os caracteres de pontuação e acentos gráficos foram excluídos, e os termos foram passados para o singular, com base nas regras de plural mais usadas no português (há exceções). Em seguida, palavras vazias comuns, conjunções, advérbios e numerais foram excluídos. As palavras restantes foram listadas por frequência de uso e inspecionadas para correção de erros de digitação. Verbos, adjetivos e substantivos dotados do mesmo significado (por exemplo: “estresse” e “estressado” e “ansiedade” e “ansioso”) foram agrupados. Feitos esses ajustes, a frequência foi recalculada, e as palavras que apareceram duas ou mais vezes foram utilizadas para a criação da nuvem de palavras. Para efeito de publicação, as palavras foram vertidas para o inglês ( Material suplementar S3 ). Todos os dados e códigos podem ser acessados mediante solicitação encaminhada ao autor designado para correspondência e com a devida aprovação pelo comitê de ética.

## RESULTADOS

### Dados demográficos

Entre os dias 3 e 10 de abril de 2020, 2.646 pesquisas foram completadas (taxa aproximada de resposta de 19%). As características demográficas da amostra encontram-se dispostas na tabela 1 .

A mediana de idade dos participantes foi 37 anos (variação de 19 a 74 anos). Entre os respondentes, 2.066 (78,1%) eram mulheres, 989 (37,4%) fizeram pós-graduação, e 1.304 (49,3%) estavam na linha de frente. A maioria dos respondentes (2.078; 78,5%) relatou contato com casos confirmados ou suspeitos de COVID-19 no trabalho, e 756 (28,6%) informaram ter modificado a rotina de trabalho em resposta à COVID-19. A alteração mais frequente foi trabalhar parcialmente em casa, de forma remota (414 pessoas, 15,6%).

### Fontes, tipos e volume de informações relacionadas à COVID-19

A maioria dos respondentes (90,1%) acessou informações sobre COVID-19 nos canais tradicionais de mídia (televisão/rádio), seguidos por WhatsApp (73,0%), boca a boca (57,0%), *e-mail* (54,4%), Workplace (47,7%), Facebook (47,2%), Instagram (40,4%), YouTube (22,1%), outros (10,8%) e Twitter (7,0%). Quando questionados sobre tipos específicos de informações que receberam, a mais frequente foi sobre o número de casos confirmados (recebida por 96,3%), seguida de perto por medidas de prevenção (94,9%), número de mortes por COVID-19 (94,4%) e sintomas da COVID-19 (91,0%). Informações sobre possíveis tratamentos foram relatadas por 72,9%, enquanto 8,8% descreveram outros tipos de informações, e 0,2% disse não ter recebido nenhuma informação sobre COVID-19.

Em seguida, buscou-se caracterizar o volume de informações relacionadas à COVID-19 acessadas pelos respondentes e eventuais mudanças de comportamento de uso de mídias sociais durante a pandemia. Aproximadamente 44,4% dos respondentes relataram acesso excessivo (escores acima de 75 em uma escala de zero a cem; materiais suplementares S1 e S2 ) a informações relacionadas à COVID-19. Além disso, a maioria dos participantes (67,6%) relatou aumento do consumo diário de mídia (aumento médio de 2,46 horas, desvio-padrão de 4,35 horas) em relação ao acesso regular antes da pandemia. Apesar do aumento médio do consumo diário de mídias sociais, 45,5% dos participantes relataram redução do acesso a informações relacionadas à COVID-19 na semana anterior, principalmente devido à redundância das informações (77,7%), mas também por cansaço (59,54%) e medo do teor das informações (19,8%), entre outros motivos (14,0%).

### Sentimentos relacionados às demandas de processamento de informações durante a pandemia

No que se refere às percepções e aos sentimentos associados às informações relacionadas à COVID-19 ( Figura 1 ), muitos participantes se sentiram estressados pelo volume de informações para se manterem atualizados (sempre ou quase diariamente: 30,7%). Entretanto, 24,7% dos respondentes continuaram consumindo informações após terem atingido o próprio limite (sempre ou quase sempre). Ao serem questionados quanto à facilidade de determinar a confiabilidade das informações relacionadas à COVID-19, a maioria dos participantes respondeu que, às vezes, era fácil (43,8%). A maioria dos participantes relatou não ter dificuldade para encontrar as informações desejadas (nunca: 39,6%; pelo menos às vezes: 34,0%) e ter confiança na própria capacidade de encontrar as informações desejada na maioria das vezes (sempre ou quase diariamente: 59,7%). Após obterem as informações, os respondentes acreditaram elas que foram úteis para tomada de decisões (sempre ou quase diariamente: 57,1%). Ainda, a maioria reportou que seu cargo ou sua instituição demandaram busca de informações sobre a COVID-19 (sempre ou quase diariamente: 60,4%).

**Figura 1:** Percepções e sentimentos relacionados às informações sobre COVID-19. Frequência de respostas a cada pergunta na escala Likert, apresentada na forma de gráficos de barras horizontais cumulativos

### Queixas relacionadas ao sofrimento psicológico

Os participantes foram questionados quanto a sintomas associados ao sofrimento psicológico na semana anterior. Mais da metade dos participantes relataram ter tido dor de cabeça (57,9%), e quase metade relatou dificuldade para dormir (49,5%), entre outros sintomas ( Figura 2 A). A análise dos subgrupos de estilos de processamento diante da sobrecarga de informações revelou maior frequência de sintomas de sofrimento psicológico entre os participantes com estilo negativo em relação aos participantes com estilo positivo ( Figura 2 B; intervalos de confiança para diferenças de proporções: dor de cabeça = 10,38–18,71; dificuldade para dormir = 12,58%–21,05%; inquietação = 19,22%–27,54%; espasmos ou contrações nos olhos = 4,64%–10,99%; ausência de sintomas = −12,68%– −19,12% e outros = 1,44%–7,21%). A frequência de sintomas de sofrimento psicológico também foi maior entre participantes cujo acesso às mídias sociais aumentou durante a pandemia em relação àqueles cujo acesso diminuiu, exceto no caso das alternativas “espasmos ou contrações nos olhos” e “outros sintomas” ( Figura 2 C; intervalos de confiança para diferenças de proporções: dor de cabeça = 0,01%–8,25%; dificuldade para dormir = 6,14%–14,39%; inquietação = 6,29%–14,31%; espasmos ou contrações nos olhos = −0,79%–5,15%; ausência = −2,96%– −9,89%; outros = −1,96%–3,58%).

**Figura 2:** Prevalência de sintomas de sofrimento psicológico. (A) Gráficos de barras mostrando a frequência de sintomas entre todos os participantes, (B) frequência de sintomas de acordo com o estilo de processamento de informação (positivo e negativo) e (C) o aumento ou diminuição do tempo dispendido em mídias sociais (isto é, horas de consumo diário de mídia) na semana anterior à pesquisa em relação a antes da pandemia

No que se refere a outros sintomas relatados nas respostas dadas à pergunta aberta, as palavras mais usadas foram dor (111 vezes), ansiedade (70 vezes), falta (43 vezes), cansaço (34 vezes) e garganta (33 vezes). A nuvem de palavras contendo todos os termos usados duas vezes ou mais pode ser visualizada na figura 3 . Cabe notar que a palavra dor pode expressar “ *ache* ” e “ *pain* ” no idioma inglês, dependendo do contexto. Por exemplo, “ *headache* ” corresponde a dor de cabeça e “ *back pain* ” a dor nas costas. O mesmo se aplica à palavra “falta”, que pode se referir a “ *lack* ” como em falta de apetite (“ *lack of apetite* ”) ou “ *shortness* ”, como em falta de ar (“ *shortness of breath* ”). A nuvem contendo as palavras em inglês pode ser visualizada no material suplementar S3 .

**Figura 3:** Nuvem de palavras utilizadas na descrição de sintomas de sofrimento psicológico. Nuvem criada a partir de palavras extraídas das respostas dadas pelos respondentes no campo “outros”, quando questionados sobre os sintomas vivenciados. Palavras usadas uma única vez foram omitidas

## DISCUSSÃO

Esta pesquisa transversal *on-line* com funcionários de uma grande instituição de saúde localizada em São Paulo, Brasil, incluiu 2.646 respondentes e revelou elevação geral da exposição diária a informações relacionadas à COVID-19 veiculadas em canais tradicionais e em mídias sociais, com aumento correspondente da frequência de sintomas de sofrimento psicológico. Os participantes eram em sua maioria mulheres com mediana de idade de 37 (19 a 74) anos. A maioria dos participantes tinha escolaridade superior (graduação ou superior). Este estudo forneceu dados a respeito do comportamento de consumo de informações relacionadas à COVID-19 entre os profissionais da saúde durante a pandemia. Além disso, os achados sugerem que a exposição excessiva às informações e as altas demandas de processamento podem causar sofrimento psicológico.

O aumento do consumo diário de mídia relatado pela maioria dos participantes desta pesquisa é compatível com teorias de comunicação em massa, que descrevem aumento da dependência de mídias em tempos de crise e incerteza.^(^^10^^)^Além disso, a maioria dos respondentes também relatou uso de canais tradicionais de mídia, como televisão, para obter informações sobre a COVID-19. Curiosamente, o segundo canal mais usado foi o WhatsApp, um tipo de mídia social amplamente divulgado no Brasil. A exposição preferencial a canais que exibem conteúdo gráfico também pode ter papel importante na compreensão das implicações do consumo de mídia para a saúde durante surtos de doenças. No caso do atentado à bomba ocorrido na maratona de Boston em 2013, por exemplo, foram relatadas associações entre maior exposição a imagens que continham sangue e maiores níveis de estresse pós-traumático e medo do futuro 6 meses após o incidente.^(^^11^^)^Entretanto, os resultados deste estudo mostraram que proporção significativa (45,5%) de participantes relatou diminuição deliberada do acesso às informações relacionadas à COVID-19 por motivos como redundância de informações e cansaço, sugerindo importante papel das estratégias de autocontrole na regulação do comportamento de consumo de informações.^(^^12^^)^

As respostas relacionadas aos possíveis sinais de sobrecarga de informações associada à pandemia indicaram que aproximadamente um terço dos respondentes se sentiram estressados quase todos os dias ou sempre, devido à quantidade de informações para acompanhar. Do total de respondentes, mais da metade relatou ter tido pelo menos um sintoma de sofrimento psicológico (dor de cabeça, espasmos ou contrações nos olhos, inquietação ou dificuldade para dormir) e a frequência de sintomas foi maior entre os participantes com viés negativo em relação à demanda excessiva de processamento de informações. Dor e ansiedade foram as palavras mais empregadas pelos participantes nas respostas à pergunta aberta sobre sintomas de sofrimento psicológico. Além disso, esses sintomas foram mais frequentes entre os participantes que relataram maior uso de mídia social. A ampla cobertura midiática e outros fatores, como aumento duradouro do número de casos de infecção por COVID-19, grande sobrecarga de trabalho e baixa disponibilidade de equipamento de proteção individual, foram apontados como possíveis responsáveis pelos desfechos de saúde mental entre os profissionais da saúde chineses durante a crise da COVID-19. Índices mais elevados de insônia foram observados entre os profissionais da área médica que dedicaram mais de 5 horas à leitura de notícias relacionadas à COVID-19.^(^^13^^)^Além disso, a alta prevalência de problemas de saúde mental foi positivamente associada à exposição à mídia social na população chinesa,^(^^14^^)^e o maior tempo dispendido na leitura de informações relacionadas à COVID-19 foi associado a níveis mais elevados de ansiedade entre os russos.^(^^15^^)^Esses achados reforçam a hipótese de que a exposição repetida à mídia durante o surto vigente de coronavírus pode ter relação com o sofrimento psicológico.^(^^3^^)^Entretanto, os achados desta pesquisa indicam que as diferenças individuais de estilo de processamento diante da sobrecarga de informações podem modular o sofrimento psicológico associado à exposição às informações, dada a maior frequência de sintomas relacionados entre os participantes que perceberam o excesso de informações como estressante e/ou complexo.

Embora despender mais tempo no consumo de informações relacionadas à COVID-19 tenha sido associado com sofrimento psicológico, ter informações precisas e atualizadas sobre questões de saúde parece ser um fator protetor.^(^^16^^)^Neste estudo, foi observado que a maioria dos participantes nunca tinha dificuldade de encontrar as informações desejadas (39,6%), embora percentagem significativa tenha relatado dificuldades pelo menos algumas vezes (34,0%). Além disso, o fato de a maioria dos participantes ter declarado que às vezes era fácil determinar a confiabilidade das informações indica que, algumas vezes, isso não era fácil, mesmo para funcionários de uma grande instituição de saúde. Portanto, o potencial efeito protetor pode ter sido atenuado nesta amostra.

Dentre as limitações deste estudo, destacam-se o emprego de uma amostra obtida mediante amostragem por conveniência e composta majoritariamente por mulheres (as mulheres são maioria entre os funcionários da instituição de saúde selecionada), a baixa taxa de resposta (19%) e o desconhecimento da validade psicométrica da pesquisa (o questionário empregado ainda não foi validado). Outra limitação diz respeito ao fato de que os envolvidos mudaram seus hábitos de acesso à mídia ao longo da pandemia, como mostra a percentagem de respondentes que relatou diminuição de acesso na semana da pesquisa. Cabe notar que a pesquisa foi disponibilizada 23 dias após o anúncio da pandemia pela Organização Mundial de Saúde, ocorrido em 11 de março de 2020, e 10 dias após a implementação oficial das medidas de quarentena em São Paulo (24 de março de 2020). Portanto, novos estudos se fazem necessários para investigar mudanças de comportamento de acesso à mídia durante a pandemia. Entretanto, apesar das limitações descritas, os achados desta pesquisa podem contribuir para a elaboração de políticas de comunicação e intervenções destinadas a promover o bem-estar psicológico no ambiente de trabalho.

## CONCLUSÃO

Este estudo forneceu uma descrição do padrão de consumo de informações relacionadas à doença causada pelo novo coronavírus 2019 durante a pandemia, por parte dos profissionais do setor de saúde. Os achados sugerem que a exposição excessiva às informações e a alta demanda de processamento podem acarretar sintomas de sofrimento psicológico.
